# A national study of choanal atresia in tertiary care centers in Canada – part I: clinical presentation

**DOI:** 10.1186/s40463-021-00517-x

**Published:** 2021-07-12

**Authors:** Josee Paradis, Agnieszka Dzioba, Hamdy El-Hakim, Paul Hong, Frederick K. Kozak, Lily H. P. Nguyen, Demitri Perera, Evan Jon Propst, Jennifer M. Siu, Monika Wojtera, Murad Husein, National Choanal Atresia Study Working Group

**Affiliations:** 1 Department of Otolaryngology- Head and Neck Surgery, Children’s Hospital at London Health Sciences Centre, London, ON Canada; 2 Otolaryngology-Head and Neck Surgery, Schulich School of Medicine and Dentistry, Western University, London, ON Canada; 3 Division of Pediatric Surgery and Otolaryngology Head and Neck Surgery, Departments of Surgery and Pediatrics, The Stollery Children’s Hospital, University of Alberta Hospital, Edmonton, AB Canada; 4 IWK Health Centre, Halifax, NS Canada; 5 Division of Otolaryngology-Head and Neck Surgery, Department of Surgery, Dalhousie University, Halifax, NS Canada; 6 Faculty of Medicine, University of British Columbia, Vancouver, BC Canada; 7 Division of Pediatric Otolaryngology-Head and Neck Surgery, BC Children’s Hospital, Vancouver, BC Canada; 8 Department of Otolaryngology-Head and Neck Surgery, McGill University, Montreal, Canada; 9 Institute for Health Science Education, McGill University, Montreal, Canada; 10 Department of Pediatric Surgery, Montreal Children’s Hospital, Montreal, Canada; 11 Faculty of Medicine, University of Queensland, Brisbane, Queensland Australia; 12 Department of Otolaryngology-Head & Neck Surgery, Hospital for Sick Children, University of Toronto, Toronto, ON Canada

**Keywords:** Choanal atresia, Clinical presentation, Family history

## Abstract

**Background:**

To evaluate the clinical presentation of choanal atresia (CA) in tertiary centers across Canada.

**Methods:**

Multi-centre case series involving six tertiary care pediatric hospitals across Canada. Retrospective chart review of patients born between 1980 and 2010 diagnosed with CA at a participating center.

**Results:**

The health charts of 215 patients (59.6% female) with CA were reviewed and included in this study. The mean age of patients at time of CA presentation was 0.4 months (range 0.1 to 7.2 months) for bilateral CA and 37.8 months (range 0.1 to 164.1 months) for unilateral cases. The most common presenting symptoms for bilateral CA in decreasing order were respiratory distress (96.4%), feeding difficulties (68.2%), and rhinorrhea (65.5%), and for unilateral cases in decreasing order were rhinorrhea (92.0%), feeding difficulties (24.7%), and respiratory distress (18.0%). For the majority of patients (73.2%), the obstruction comprised mixed bony and membranous tissue, with only 10.5% presenting with a purely membranous obstruction. Familial history of CA was confirmed in only 3.3% of cases. One half of patients with CA presented with one or more associated anomalies and 30.6% had a syndrome.

**Conclusions:**

The present investigation is the first national multi-institutional study evaluating the clinical presentation of CA over three decades. The present cohort of CA patients presented with a breadth of co-morbidities with highly variable presentations, with bilateral cases being more severely affected than unilateral cases. Further investigation into hereditary linkages to CA development is warranted.

**Graphical abstract:**

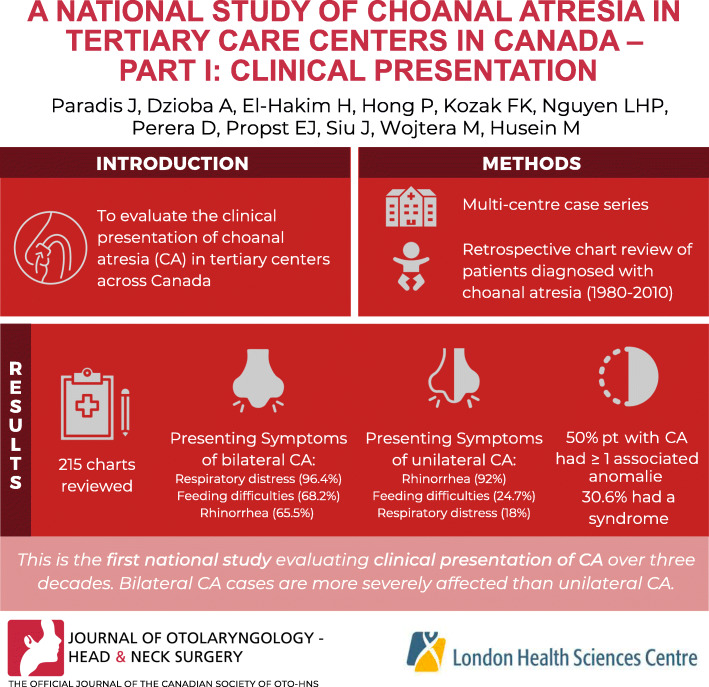

## Background

Choanal atresia (CA) is a congenital condition resulting in obstruction of the posterior nasal passage(s), known as the choana, that may be bony, membranous or mixed bony-membranous. The incidence of this uncommon entity is approximately one in 5000 to 8000 live births [1, 2]. The clinical presentation of CA may include rhinorrhea, respiratory distress (particularly in the presence of bilateral CA), feeding difficulties and nasal obstruction. Approximately one half of patients with CA present with associated anomalies and/or syndromes, with the most common syndrome being CHARGE [3–5]. Although a breadth of literature regarding the pathogenesis of CA exists, risk factors for development of CA still need to be elucidated [6].

To date, no agreement regarding best practices for management of CA exist. Ongoing controversy regarding best practices for clinical management of CA lies, in part, in the rarity of the disorder and consequently the limited level of evidence available in published studies on this subject matter. The literature often reports case series of single surgeon or single institution experiences, involving small sample sizes [7–9], with few studies in the English-language literature reporting outcomes on samples sizes greater than 30 patients [9–15]. The aim of the current investigation is to explore outstanding controversies surrounding the presentation, diagnosis and family history of CA, using a multi-institutional approach. The Canadian landscape lends a unique setting as all CA repairs are performed in an academic setting. In a two-part investigation, this national study provides a comprehensive review of the clinical presentation (Part I) and management (Part II) of a large sample of patients with CA treated at tertiary care centres across Canada. The present paper reports on the clinical presentation of CA. Specifically, the following outcomes were explored: presentation and diagnosis of CA, and family history.

## Methods

Pediatric otolaryngologists practicing in tertiary care centers across Canada were invited, via phone or email, to participate in the study. Of the nine centers who were contacted, six centers (Western University in London, University of Toronto in Toronto, University of Alberta in Edmonton, University of British Columbia in Vancouver, McGill University in Montreal and Dalhousie University in Halifax) agreed to participate in the chart review. Representatives of each participating center formed the Canadian Choanal Atresia working group (in acknowledgment). Health charts of patients born between 1980 and 2010 diagnosed with CA who underwent treatment at participating centers were included in this study. Patients born before 1980 or after 2010 or patients who did not receive a definitive diagnosis of CA were excluded from the study. A standardized checklist was completed for each patient presenting with CA who met study inclusion/exclusion criteria. The following variables were collected from patient chart review: incidence of CA in Canada, presenting signs and symptoms of CA, requirement of intubation, reports of CT scans, use of suction catheter to aid in diagnosis, characteristics of CA, associated syndromes and co-morbidities, family history, and, a comparison of CA characteristics for unilateral versus bilateral cases. Ethical approval for this study was obtained from the Health Research Ethics Boards at each participating center.

### Data analysis

Descriptive analyses including frequencies, means and standard deviations of study outcomes were undertaken. In addition to descriptive statistics, results of chi-square tests/Fisher’s exact tests, and independent samples t-tests were reported where appropriate. The incidence rate of CA in Canada was estimated based on the following calculation: total number of births in provinces in Canada that were well represented by participating centers between 1980 and 2010 [16, 17], divided by the number of patients seen at participating centers that serviced those provinces during that time period: New Brunswick, Prince Edward Island, and Nova Scotia (serviced by Dalhousie University in Halifax); British Columbia (serviced by University of British Columbia in Vancouver), and Ontario (serviced by Western University in London and University of Toronto in Toronto). Montreal and Edmonton’s data were excluded from incidence calculation as these centers did not adequately represent case counts for the provinces of Quebec and Alberta.. Statistical analyses were conducted using SPSS (IBM Corp. Released 2017. IBM SPSS Statistics for Windows, Version 25.0. Armonk, NY: IBM Corp). An alpha level of .05 was set to determine statistical significance.

## Results

Two-hundred and fifteen patients across the six participating centers [London (*n* = 26), Toronto (*n* = 83), Edmonton (*n* = 17), Vancouver (*n* = 60), Montreal (*n* = 11), Halifax (*n* = 18)] met the study inclusion criteria and were included in this national study. Health charts of 215 patients were reviewed. One-hundred and twenty-seven patients (59.6%) were female and 88 (40.9%) were male. The mean age of patients at time of CA presentation was 0.4 months (range 0.1 to 7.2 months) for bilateral CA and 37.8 months (range 0.1 to 164.1 months) for unilateral cases. Patients were born pre-term on average [mean (SD) for gestational age was 35.0 (9.0) weeks].

### Incidence of CA in Canada

One-hundred and eighty-seven cases of CA were reported across Dalhousie University (*n* = 18), University of British Columbia (*n* = 60), Western University (*n* = 26), and University of Toronto (*n* = 83) over the 30-year study period. There were 6,211,520 total births in New Brunswick, Prince Edward Island, Nova Scotia, British Columbia and Ontario between 1980 and 2010 [16, 17]. Diving the total births, 6,211,520 by the estimated cases of CA, 187, resulted in an estimated incidence rate of 1 in 33,217. As such, we approximate the incidence rate for CA in Canada to be between 1 in 30,000 to 40,000.

### Presenting signs and symptoms

Patients presented with varying symptom to their respective paediatric otolaryngology clinic, or, for bilateral cases, from inpatient referral from the neonatal intensive care unit (NICU). The most common presenting symptom was rhinorrhea (bilateral: 65.5%, unilateral: 92.0%), with the majority of unilateral cases being on the right side (65.5%). Other symptoms included respiratory distress (bilateral: 96.4%; unilateral: 18.0%; entire cohort: 55.8%), and feeding difficulties (bilateral: 68.2%, unilateral: 24.7%; entire cohort: 43.7%). For those presenting with respiratory distress (*n* = 96), intubation was required in 65 cases (67.7%; 60 bilateral, 5 unilateral). On clinical investigation, clinicians were unable to pass a suctioncatheter through the nasal passage in 87 patients (85.3%; 52 bilateral, 35 unilateral), 58.6% of which failed the suction catheter test on both sides of the nasal passages (49 bilateral, 2 unilateral). Table 1 displays details of the presenting signs and symptoms for the entire study cohort, and for bilateral and unilateral CA.

**Table 1:** Presenting signs and symptoms of choanal atresia

Symptom	Category	Unilateral CA No. (%)	Bilateral CA No. (%)	Total No. (%)
Rhinorrhea	Yes	92 (92.0)	36 (65.5)	128 (82.6)
	No	8 (8.0)	19 (34.5)	27 (17.4)
If yes, predominant side	Right	55/92 (59.9)	2/36 (5.6)	57/128 (44.5)
	Left	27/92 (29.3)	1/36 (2.8)	28/128 (21.9)
	Both	5/92 (5.4)	30/36 (83.3)	35/128 (27.3)
	Unknown	5/92 (5.4)	3/36 (8.3)	8/128 (6.3)
Feeding difficulties	Yes	21 (24.7)	45 (68.2)	66 (43.7)
	No	64 (75.3)	21 (31.8)	85 (56.3)
Respiratory Distress	Yes	16 (18.0)	80 (96.4)	96 (55.8)
	No	73 (82.0)	3 (3.6)	76 (44.2)
If yes, intubation required	Yes	5/16 (31.2)	60/80 (75.0)	65/96 (67.7)
	No	7/16 (43.8)	14/80 (17.5)	21/96 (21.9)
	Unknown	4/16 (25.0)	6/80 (7.5)	10/96 (10.4)
Unable to pass suction catheter	Yes	35 (77.8)	52 (91.2)	87 (85.3)
	No	10 (22.2)	5 (8.8)	15 (14.7)
If yes, side	Right	21/35 (60.0)	1/52 (1.9)	22/87 (25.3)
	Left	12/35 (34.3)	0/52 (0)	12/87 (13.8)
	Both	2/35 (5.7)	49/52 (94.2)	51/87 (58.6)
	Unknown	0/35 (0)	2/52 (3.9)	2/87 (2.3)
CT Scan Identifying CA	Yes	90 (79.6)	63 (85.1)	153 (81.8)
	No	23 (20.4)	11 (14.9)	34 (18.2)

### Choanal atresia characteristics and associations

Table 2 displays CA characteristics and associations for the study cohort. Computerized tomography (CT) scans confirming a diagnosis of CA were performed in 153 cases (81.8%). For the majority of patients (*n* = 139; 73.2%), the obstruction comprised mixed bony and membranous tissue, while only 20 cases (10.5%) presented with a purely membranous obstruction. Choanal atresia was bilateral in 43.3% of cases (*n* = 93). Approximately one half of patients with CA [*n* = 112 (52.6%); bilateral: *n* = 65 (69.9%); unilateral: *n* = 47 (39.2%)] presented with one or more associated anomaly. Sixty patients (30.6%) were identified with a syndrome; of the 93 patients with bilateral CA, 37 (45.1%) presented with a syndrome, and, of the 122 patients who had unilateral CA, 23 (20.2%) presented with a syndrome. Thirty-five patients (25 bilateral, 10 unilateral) had confirmed CHARGE syndrome. Table 3 displays CHARGE associations and other anomalies for the entire study cohort and the subgroup of patients who had a confirmed CHARGE syndrome.

**Table 2:** Choanal Atresia Characteristics and Associations

Variable	Category	Unilateral CA No. (%)	Bilateral CA No. (%)	Total No. (%)
Type of CA	Membranous	10 (9.0)	10 (12.7)	20 (10.5)
	Bony	16 (14.4)	15 (19.0)	31 (16.3)
	Mixed	85 (76.6)	54 (68.4)	139 (73.2)
Laterality of CA	Right	80 (65.5)	–	80 (37.2)
	Left	42 (34.4)	–	42 (19.5)
	Bilateral	–	93 (100)	93 (43.3)
Associated anomalies	Yes	47 (39.2)	65 (69.9)	112 (52.6)
	No	73 (60.8)	28 (30.1)	101 (47.4)
Associated syndrome	Yes	23 (20.2)	37 (45.1)	60 (30.6)
	No	91 (79.8)	45 (54.9)	136 (69.4)
If yes, CHARGE	Yes	10/23 (43.5)	25/37 (67.6)	35/60 (58.3)
	No	11/23 (47.8)	10/37 (27.0)	21/60 (35.0)
	Unknown	2/23 (8.7)	2/37 (5.4)	4/60 (6.7)
If yes, other syndrome	Yes	14/23 (60.9)	16/37 (43.2)	30/60 (50.0)
	No	5/23 (21.7)	11/37 (29.7)	16/60 (26.7)
	Unknown	4/23 (17.4)	10/37 (27.0)	14/60 (23.3)
Abnormal karyotype	Yes	7 (9.0)	6 (8.0)	13 (8.5)
	No	71 (91.0)	69 (92.0)	140 (91.5)

**Table 3:** CHARGE Associations and Other Anomalies

Variable	Category	CHARGE group (***n*** = 35) No. (%)	Total (***n*** = 215) No. (%)
(C) Coloboma and/or micropthalmia	Coloboma	10 (28.6)	20 (9.3)
	Micropthalmia	2 (5.7)	4 (1.9)
	Visual impairment	3 (8.6)	3 (1.4)
(H) Heart defects	VSD	14 (40.0)	20 (9.3)
	ASD	14 (40.0)	17 (7.9)
	PDA	4 (11.4)	12 (5.6)
	patent foramen ovale	2 (5.7)	5 (2.3)
	Aortic arch anomaly	0 (0)	2 (0.9)
	Tetralogy of the heart	1 (2.9)	2 (0.9)
	Other heart anomalies	7 (20.0)	12 (5.6)
(A) Atresia of the choana	Choanal atresia	35 (100)	215 (100)
(R) Retarded growth and development	Retarded growth	4 (11.4)	6 (2.8)
	Developmental delay	5 (14.3)	7 (3.3)
	CNS anomalies	6 (17.1)	18 (8.4)
(G) Genital hypoplasia	Undescended testes	6 (17.1)	6 (2.8)
	Micropenis	3 (8.6)	3 (1.4)
	Labial adhesions	1 (2.9)	1 (0.5)
	Undefined genital anomalies	1 (2.9)	1 (0.5)
(E) Ear anomalies and/or hearing loss	Ear anomalies	14 (40.0)	16 (7.4)
	SNHL	13 (37.1)	19 (8.8)
	Conductive hearing loss	1 (2.9)	1 (0.5)
Other dysmorphic features	Dysmorphic facial features	11 (31.4)	28 (13.0)
	Strabismus/Proptosis	0 (0)	6 (2.8)
	Micro/retrognathia	2 (5.7)	9 (4.2)
	Digital anomalies	2 (5.7)	12 (5.6)
	Upper limb defects	1 (2.9)	3 (1.4)
	Foot anomalies	2 (5.7)	5 (2.3)
	Undefined dysmorphic features	7 (20.0)	15 (7.0)
Palatal Anomalies	Cleft lip and/or palate	2 (5.7)	20 (9.3)
	High-arched palate	1 (2.9)	2 (0.9)
Airway abnormalities	Tracheoesophageal fistula	3 (8.6)	5 (2.3)
	Tracheomalacia	3 (8.6)	5 (2.3)
	Tracheal stenosis	0 (0)	3 (1.4)
	Laryngeal abnormalities	4 (11.4)	17 (7.9)
	Obstructive sleep apnea	1 (2.9)	2 (0.9)
Other Anomalies/Conditions	Facial nerve palsy	3 (8.6)	4 (1.9)
	Hypothalamo-hypophyseal	2 (5.7)	3 (1.4)
	Urinary tract	9 (25.7)	19 (8.8)
	Gastro-intestinal	7 (20.0)	14 (6.5)
	Skeletal (excluding limb defects)	3 (8.6)	9 (4.2)
	Pulmonary	2 (5.7)	3 (1.4)

*Note. VSD* Ventricular septal defect, *ASD* Atrial septal defect, *PDA* Patent ductus arteriosus, *CNS* Central nervous system, *SNHL* Sensorineural hearing loss

For the CHARGE subgroup, the prevalence of CHARGE-related anomalies ranged from 5.7 to 40.0%, with a high prevalence of coloboma, heart defects, ear anomalies and sensorineural hearing loss (see Table 3). Central nervous system (CNS) anomalies, laryngeal abnormalities, urinary tract conditions, skeletal anomalies and pulmonary conditions were present in both the CHARGE subgroup and entire cohort (see Table 3). The CNS anomalies, in decreasing frequency: hydrocephalus, corpus callosum agenesis, corpus callosum dysgenesis, brain hemorrhage, chiari malformation, subdural hygromas, ventriculomegaly and benign rolandic epilepsy. Laryngeal abnormalities included, in decreasing frequency: glottic edema, vocal cord paralysis, subglottic stenosis, laryngomalacia, and hypotonic larynx. Urinary tract conditions included, in decreasing frequency: hydronephrosis, vesiculourethral reflux, duplicate kidney, distal ureterocele, left pelvic kidney, horseshoe kidney, and pelvicalyceal dilation. Gastrointestinal conditions included, in decreasing frequency: imperforate anus, duodenal atresia, gastroesophageal reflux disease (GERD), inguinal hernia, and Hirschsprung’s disease. Skeletal anomalies (excluding limb defects) included scoliosis, congenital hip dislocation, bifid ribs and fused vertebrae. Finally, pulmonary conditions included chronic lung disease, asthma and pneumothorax.

Of available syndrome information in patient charts, thirty patients (50.0%) had been diagnosed with other syndromes, and six of these patients were diagnosed with a syndrome in addition to CHARGE. Syndromes included Down syndrome (*n* = 7), Pierre Robin sequence (*n* = 2), Treacher-Collins Syndrome (*n* = 2), fetal alcohol syndrome (*n* = 2), Apert’s syndrome (*n* = 1), Autism (*n* = 1), Cerebral palsy (*n* = 1), Clefting syndrome (*n* = 1), Cornelia de Lange (*n* = 1), Crouzon’s (*n* = 1), Di George syndrome (*n* = 1), Duane-radial ray syndrome (*n* = 1), Goldenhar syndrome (*n* = 1), Kabuki syndrome (*n* = 1), Moebius syndrome (*n* = 1), Pallister-Hall syndrome (*n* = 1), Pfeiffer syndrome (*n* = 1), Riley-day syndrome (*n* = 1), Ring chromosome 18 (*n* = 1), Turner Syndrome (*n* = 1) and undetermined genetic syndrome (*n* = 1).

Only thirteen patients (8.5%) had a confirmed abnormal karyotype. Varied karyotype abnormalities were found including: 45, X0; 45, XX; 46, XX, (9p+); 47, XY; Partial trisomy 15; Trisomy 18; Trisomy 21; Trisomy 22; Ring chromosome 18; Robertsonian translocation between 14 & 22, 14 & 15; and, SALL4 gene mutation.

### Family history

Table 4 displays the frequency statistics of familial history for CA. Seven patients (3.3%), all of which had bilateral CA, had a family history of CA. In three patients, only the father had CA; in two patients, the mother and a sibling had CA; in one patient, only a sibling had CA; and, in one patient, only the mother had CA. All three siblings with CA had CHARGE syndrome. Laterality of CA was unknown in all cases of familial CA. Other noted relevant medical history of family of patients with CA included two cases of bilateral cleft lip and palate, and single cases of abnormal ears, club foot, deviated septum, duplex kidney, hearing loss, heart defect, and Down syndrome.

**Table 4:** Familial History (*n* = 215)

Variable	Category	No. (%)
Siblings with CA	Yes	3 (2.0)
	No	146 (98.0)
Father with CA	Yes	3 (2.1)
	No	137 (97.9)
Mother with CA	Yes	3 (2.1)
	No	139 (97.9)
Maternal Age (Years)	Mean (SD)	28.5 (5.3)

### Bilateral versus unilateral CA

Table 5 displays CA characteristics, comparing bilateral versus unilateral CA. The mean age at presentation of CA was 0.4 months in bilateral cases and 37.8 months in unilateral cases. Individuals with bilateral CA were significantly more likely to present with an associated anomaly [(69.9% vs 39.2%), χ^2^(1) = 18.8, *p* < .001] and were also significantly more likely to present with a syndrome [(45.1% vs. 20.2%), χ^2^(1) = 12.9, *p* < .001] than individuals with unilateral CA. Respiratory distress was a predominant presenting symptom in bilateral CA cases (96.4%) and occurred in only 18.0% of unilateral cases [χ^2^ (1) = 105.2, *p* < .001]. Rhinorrhea was a predominant symptom in unilateral cases and, also in bilateral cases but statistically significantly less [(92.0% vs 65.5%), χ^2^(1) = 17.1, *p* < .001].

**Table 5:** Comparison of bilateral versus unilateral choanal atresia

Variable	Unilateral (***n*** = 122) No. (%)	Bilateral (***n*** = 93) No. (%)	***P***-value
Mean (SD) age at presentation (months)	37.8 (46.5)	0.4 (1.4)	<.001
Associated Anomaly	47 (39.2)	65 (69.9)	<.001
Associated Syndrome	23 (20.2)	37 (45.1)	<.001
Respiratory Distress	16 (18.0)	80 (96.4)	<.001
Rhinorrhea	92 (92.0)	36 (65.5)	<.001
Feeding difficulties	21 (24.7)	45 (68.2)	<.001

*Note*. *p*-values represent results of an independent samples t-test for the age at presentation variable and results of chi-square tests for the remaining variables

## Discussion

The present investigation explored the clinical presentation of CA over a 30-year period (1980 to 2010). To address some of the limitations of previous studies that were mostly single surgeon or single institution case series, resulting in small sample sizes from which to infer conclusions, this national study took a multi-institutional approach. This is the first national multi-institutional study reporting on a large sample size of 215 patients with CA, providing stronger empirical support to help elucidate some of the outstanding controversies related to the clinical manifestation of this condition. This study will contribute to the body of knowledge on clinical presentation of CA, particularly relative to CA characteristics and associations, diagnosis, and familial factors.

### Incidence of CA in Canada

Based on the synthesis of cases of patients with CA reviewed in this present investigation, the incidence rate for CA in Canada was estimated to be between one in 30,000 to 40,000. As such, the present investigation found the incidence of CA to be much lower than incidence rates of approximately one in 5000 to 8000 live births commonly reported in the literature [1, 2].

### Presenting signs and symptoms

Signs and symptoms of CA are well known in the literature, however, the specific rates of symptoms are not well reported. In the present study, presenting symptoms included rhinorrhea in 82.6% of cases and respiratory distress in 55.8% of cases, of which 67.7% required intubation. In addition, a significant number (43.7%) also presented with feeding difficulties. Patients who presented with bilateral CA had statistically significantly higher rates of symptoms than unilateral CA. Most patients with bilateral CA presented with respiratory distress (96.4%) and many presented with rhinorrhea (65.5%) and feeding difficulties (68.2%).

Persistent symptoms of rhinorrhea, respiratory distress, and/or nasal obstruction usually prompt further evaluation of the nasal and oral cavity. Inability to pass a suction catheter through the nasal passages usually prompts a more thorough evaluation of the anatomy of the nasopharynx, with the use of nasal endoscopy and, possible CT scan [18] to confirm diagnosis of CA. Of charts with available information, 85.3% of patients with CA did fail the suction catheter test, while 14.7% did not fail the test [10 (22.2%) of unilateral cases; 5 (8.8%) of bilateral cases]. 81.8% of cases had a CT scan in the present investigation to either confirm the diagnosis or further characterize the type of CA and, was not utilized in 18.2% of cases as a diagnostic measure.

### Choanal atresia characteristics and associations

As is reported in most case series, a predominance of females over males (59.1% female), and unilateral over bilateral (56.7% unilateral) cases were observed in the present study. Furthermore, our findings confirm previous investigations indicating one half of patients with CA present with an anomaly, and that the most common syndrome associated with CA is CHARGE [10, 18]. Patients with bilateral CA were twice as likely to present with an associated anomaly or syndrome than patients with unilateral CA in the present study.

Based on CT scan review of 47 scans from the literature and 16 from the authors’ clinical experience with CA, Brown et al. (1996) found that 29% of cases were pure bony, and 71% were mixed bony-membranous, with no cases of pure membranous being reported in this small case series [19]. Compared to findings of Brown et al. (1996) [19], in the present investigation, similar prevalence of mixed bony-membranous CA (73.2%), somewhat lower prevalence of bony CA (16.3%) and a 10.5% rate of purely membranous CA was found. Findings of the present investigation were more in line with findings of Newman et al. (2013) who reported 15, 77 and 8% as the frequency distribution for bony, mixed bony-membranous, and membranous, respectively, for their 39 cases of CA reviewed [10].

In the present study, the study cohort presented with a variety of anomalies affecting varied bodily systems including urinary, skeletal, pulmonary, gastro-intestinal, airway abnormalities, palatal anomalies and CHARGE-associations including coloboma, heart defects, retarded growth and development, genital hypoplasia, and ear anomalies. Although the majority of cases of CHARGE-associations belonged to the CHARGE-syndrome subgroup, with the exception of genital hypoplasia, all other CHARGE associations (i.e., coloboma, heart defects, developmental delay and ear anomalies) were found in some patients who belonged to the non-CHARGE syndrome subgroup as well. This finding may indicate unconfirmed cases of CHARGE-syndrome or a natural variability in the clinical profile of CA. Given the varied anomalies present in CA and its association with CHARGE, a CHARGE workup may be warranted with a diagnosis of bilateral CA.

### Familial factors

The hereditary aspects of CA have not been clearly delineated to date and are likely multifactorial [20]. In the present study, 3 mothers, 3 fathers, and 3 siblings of individuals with bilateral CA presented with CA themselves; in 3 cases, only the father has CA; in two cases, a mother and sibling had CA; in one case, only the sibling had CA; and in one case, only the mother had CA. All seven cases of bilateral CA with family history of CA were associated with an anomaly and/or syndrome; three of these cases had CHARGE syndrome. These findings suggest a hereditary link to CA but only in a small percentage (3.3%) of cases.

### Study limitations

This study was limited by its retrospective nature and missing data. Furthermore, three tertiary care centres in Canada did not participate in the present investigation, limiting the generalizability of study findings.

## Conclusions

This retrospective multi-center investigation reported on over 30-years experience of patients with CA across tertiary care centers in Canada. In line with previous literature, our study cohort presented with a breadth of co-morbidities and variability in presentation, with over one half presenting with one or more anomalies. Observed findings indicated limited hereditary linkages. Health practitioners involved in the care of patients with CA should investigate the presence of multiple anomalies. Future research investigating hereditary linkages and CA development is warranted.

## Data Availability

The datasets used and/or analyzed during the current study are available from the corresponding author on reasonable request.

## Abbreviations

CA: Choanal atresia
CHARGE: Coloboma, heart defects, atresia choanae, growth retardation, genital abnormalities, and ear abnormalities
GERD: Gastroesophageal Reflux Disease
CNS: Central nervous systmem
BHCG: Elevated beta-human chorionic gonadotropin
